# Serious safety events in rituximab‐treated multiple sclerosis and related disorders

**DOI:** 10.1002/acn3.51136

**Published:** 2020-08-06

**Authors:** Brandi L. Vollmer, Asya I. Wallach, John R. Corboy, Karolina Dubovskaya, Enrique Alvarez, Ilya Kister

**Affiliations:** ^1^ Rocky Mountain Multiple Sclerosis Center, Department of Neurology University of Colorado Aurora Colorado; ^2^ NYU Grossman School of Medicine, Department of Neurology New York University School of Medicine New York City New York

## Abstract

**Introduction:**

Studies investigating rates and risk factors for serious safety events (SSEs) during rituximab treatment of multiple sclerosis (MS), neuromyelitis optica spectrum disorders (NMOSD), and related disorders are limited.

**Methods:**

Rituximab‐treated patients with MS, NMOSD, or related disorders at the Rocky Mountain and New York University MS Care Centers were included. The follow‐up period was defined as the time from the initial dose of rituximab up to 12 months of last dose of rituximab or ocrelizumab (in patients who switched). Clinician‐reported and laboratory data were retrospectively collected from electronic medical records.

**Results:**

One‐thousand patients were included comprising 907 MS, 77 NMOSD, and 16 related disorders. Patients had a mean follow‐up of 31.1 months and a mean cumulative rituximab dose of 4012 mg. Of the 169 patients who switched to ocrelizumab, the mean ocrelizumab dose was 1141 mg. Crude incidence rate per 1000 person‐years (PY) for lymphopenia was 19.2, neutropenia 5.6, and hypogammaglobulinemia 17.8. Infections resulting in either hospitalization, IV antibiotics, or using antibiotics ≥14 days occurred at a rate of 38.6/1000 PY. Risk factors for infection were duration of therapy, male gender, increased disability, prior exposure to immunosuppression/chemotherapy, lymphopenia, and hypogammaglobulinemia. Particularly, wheelchair‐bound patients had 8.56‐fold increased odds of infections. Crude incidence rates of malignant cancer were 3.5, new autoimmune disease 2.3, thromboembolic event 3.1, and mortality of 5.4 per 1000 PY.

**Interpretation:**

Rates of SSEs in patients with MS, NMOSD, and related disorders were low. Through properly assessing risk:benefit of B‐cell depleting therapy in neuroinflammatory disorders and continual monitoring, clinicians may decrease the risk of serious infections.

## Introduction

CD20‐positive B‐cells play an essential role in the pathogenesis of multiple sclerosis (MS), neuromyelitis optica spectrum disorder (NMOSD), and other inflammatory disorders of the central nervous system.^1^, ^2^ Ocrelizumab is currently the only FDA‐approved anti‐CD20‐cell depleting therapy for MS. However, even prior to ocrelizumab approval in 2017, rituximab, a similar anti‐CD20 therapy, had been used off‐label for the treatment of MS and NMOSD. Thus, longer follow‐up data are available on rituximab‐treated neurologic patients than ocrelizumab‐treated patients.

While randomized clinical trials and real‐world studies have shown rituximab to be largely safe in MS and NMOSD, concerns have been raised about rare serious adverse events (SAE) and high‐grade lymphopenias and neutropenias.^3^, ^4^, ^5^, ^6^, ^7^, ^8^, ^9^ Infections were the most common SAE reported in phase 2 clinical trials of rituximab for MS, but none were grade 4.^3^, ^4^ Additionally, lymphopenia, neutropenia, and hypogammaglobulinemia have been reported with rituximab use in MS and NMOSD patients. Although these conditions commonly may not meet standard criteria for an SAE, they are a significant safety concern as critically low lab values may contribute to serious infections as demonstrated in rituximab‐treated rheumatoid arthritis (RA) patients.^10^ However, there is limited data on their prevalence and incidence in neurologic disorders and their impact on infection rates.^11^, ^12^, ^13^, ^14^

Previous studies investigating rituximab in the treatment of MS and NMOSD focused on therapy effectiveness and were not sufficiently powered to detect rare SAEs or changes in SAE rates with longer treatment duration.^3^, ^4^, ^5^, ^6^, ^7^, ^15^ One exception is a Swedish study conducted by Luna et al., which included infection data on 6421 MS patients, showing those on rituximab therapy to have the highest serious infection rate compared to other disease‐modifying therapies for MS.^16^ However, the estimate of SAE rates in that study was not based on individual, in‐depth chart review, which, potentially, deflated incidence rates of SAEs. Additionally, as some significant safety concerns may not meet standard criteria for SAEs, our large, two‐center study aims to address knowledge gaps through examining serious safety events (SSEs) rates and risk factors in patients with MS, NMOSD and related disorders treated long‐term with rituximab. Based on previous literature, SSEs selected for investigation were: serious infections, infusion reactions, new diagnoses of malignant cancer or autoimmune disease, serious thromboembolic events, mortality, and laboratory abnormalities (high‐grade lymphopenia, neutropenia, and hypogammaglobulinemia).

## Methods

### Patient population

Patients who (1) received ≥1 dose of rituximab prior to August 2018 at New York University MS Care Center (NYUMSCC) or the Rocky Mountain MS Center at the University of Colorado (RMMSC at CU), (2) were 18–85 years old at rituximab initiation, and (3) were diagnosed with MS, NMOSD or inflammatory disorder of the central nervous system were identified. The study was approved by the Institutional Review Boards of our respective institutions. NYUMSCC included all eligible patients. Due to limited resources, a complete review of all eligible patients at RMMSC at CU was not feasible, thus, a sample of eligible patients was randomly selected. Each patient prescribed rituximab at the Rocky Mountain MS Center prior to August 2018 (*n* = 1326) was assigned a randomly generated number. We then ordered participants by this number and consecutively reviewed participants to assess if eligibility criteria were met until a total combined sample size of 1000 was reached.

### Data collection

Clinician‐reported data were collected by thoroughly reviewing the entire medical record for the study follow‐up period for each patient. Baseline data were collected from the clinic visit closest to rituximab initiation. Outcome data were collected from rituximab start date up to 12 months post last rituximab infusion date, or last ocrelizumab infusion in cases where patients switched to ocrelizumab without a break in therapy or use of another disease‐modifying therapy. If there was no documentation of an SSE in a patient’s chart, it was considered to have not occurred for data collection and analysis purposes, with the exception of outcomes assessed by lab data. If lab data were not available, it was considered missing. At NYUMSCC and RMMSC at CU, it is standard protocol to check comprehensive blood counts with differential twice a year prior to infusions. Immunoglobulin levels were not considered standard of care at our centers throughout the study period. While the collection of immunoglobulin labs is dependent on the practitioner, current recommendations at both centers are to check immunoglobulin levels twice a year prior to infusion. Data for each center were stored on a secure server using REDcap Software. Data were de‐identified prior to being merged into a single Excel database for analysis.

### Outcome measures

Outcomes for this study were SSEs, defined as (1) lymphopenia <500 cells/mm^3^, neutropenia <1000 cells/mm^3^, or hypogammaglobulinemia with IgG values <500 mg/dL; (2) infections resulting in hospitalizations, IV antibiotics, or extended dosing antibiotics ≥14 days; (3) infusion reactions that were life‐threatening or resulted in hospitalization; (4) new diagnosis of malignant cancer; (5) new diagnosis of autoimmune disease; (6) non‐superficial thromboembolic event; (7) death. For a sub‐group of 600 randomly selected RMMSC at CU participants, all available lab data, while on rituximab/ocrelizumab were collected for IgG values, IgM values, absolute lymphocyte counts (ALC) and absolute neutrophil counts (ANC). Due to time and resource limitations, lab data could not be collected on all patients.

### Statistical analysis

Statistical analyses were conducted using SAS version 9.4. All two‐tailed *P*‐values < 0.05 were considered significant. Descriptive statistics were used to analyze baseline characteristics, follow‐up characteristics, and outcomes for the study. For outcomes, crude incidence rates per 1000 patient‐years of rituximab/ocrelizumab treatment were calculated. Missing data were most commonly seen for IgG values as regularly collected immunoglobulin levels were not considered standard of care at our centers throughout the study period. When conducting analyses, including descriptive statistics, those with missing data were excluded unless otherwise specified. Logistic regression assessed odds ratios of infection for those with/without hypogammaglobulinemia and with/without lymphopenia, adjusting for covariates selected a priori, including age, gender, disease duration, diagnosis (relapsing MS, progressive MS or other), and ambulatory disability status at baseline (no assistive device, cane use, walker use, wheelchair use, bedbound), excluding those with missing data.

For subgroup analyses, Chi‐square tests and Fisher’s exact tests were used to assess categorical data and t‐tests for continuous variables. Baseline and follow‐up characteristics were assessed for differences by the center, by diagnosis (MS vs. NMOSD), and among those who experienced an SSE compared to those who did not. Outcomes were assessed for differences by age (<55 vs. ≥55 years); ambulatory disability; history of immunosuppression/chemotherapy; and diagnosis (MS vs. NMOSD). Immunosuppression/chemotherapy use for the purpose of this study does not include MS therapies of natalizumab, dimethyl fumarate, and fingolimod. Bivariate and multivariable analyses (full model and stepwise selection) used logistic regression to assess risk factors for first serious infections.

## Results

### Baseline & follow‐up characteristics

**Figure 1:**
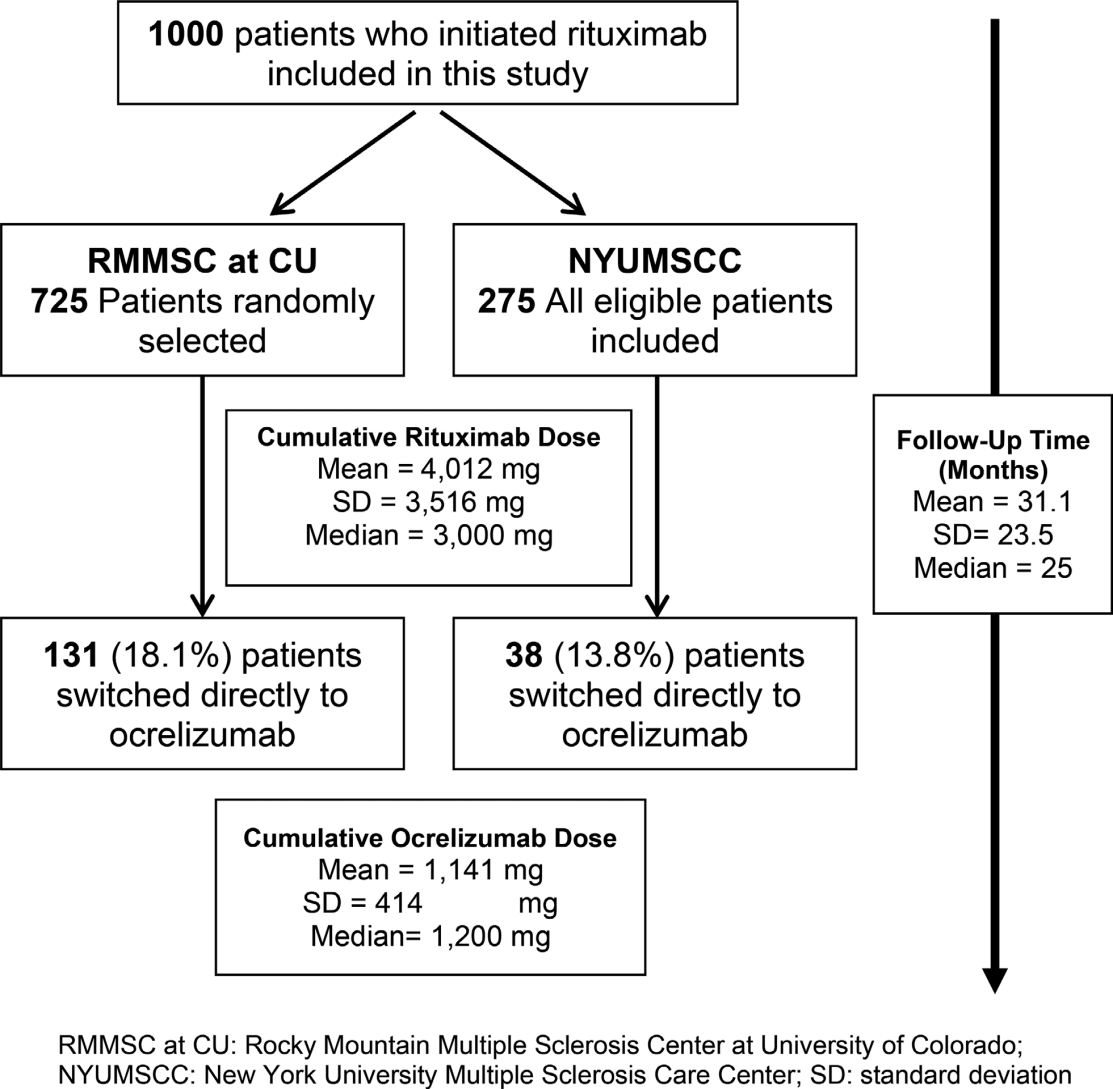
Study flow diagram.

A total of 1000 patients are included in this study, consisting of 275 treated at NYUMSCC and 725 treated at RMMSC at CU. Baseline characteristics are displayed in Table 1 overall and by diagnosis. Follow‐up duration, cumulative doses, and patients who switched to ocrelizumab for each center are shown in Figure 1 and by diagnosis in Table S1. NMOSD patients had a higher cumulative anti‐CD20 dose and longer follow‐up. For all outcomes, Table 2 demonstrates the percentage of patients who experienced an event overall and by diagnosis, as well as incidence rate per 1000 person‐years (PY) of rituximab/ocrelizumab treatment. Tables S2–S4 displays baseline and follow‐up characteristics by the study center.

**Table 1:** Baseline characteristics at first rituximab infusion and follow‐up characteristics (*N* = 1000).

Baseline characteristics	Entire study sample (*n* = 1000)		By diagnosis				
			MS (*n* = 907)		NMOSD (*n* = 77)		*P*‐value^1^
	*N* or mean	SD or %	*N* or mean	SD or %	*N* or mean	SD or %	
Age (Years, SD)	42.9	12.6	43.0	12.5	43.5	13.6	0.292
Gender – female	687	68.7%	608	67.0%	66	85.7%	**<0.001**
Race							**<0.001**
White	678	67.8%	644	71.0%	29	37.7%	
Black	139	13.9%	111	12.2%	22	28.6%	
Other	117	11.7%	93	10.3%	22	28.6%	
Unknown	66	6.6%	59	6.5%	4	5.2%	
Ethnicity							
Hispanic	107	10.7%	88	9.7%	18	23.4%	
Non‐hispanic	789	78.9%	728	80.3%	50	64.9%	
Unknown	104	10.4%	91	10.0%	9	11.7%	
Smoking status							0.074
Current smoker	131	13.2%	121	13.4%	8	10.5%	
Former smoker	266	26.7%	248	27.4%	13	17.1%	
Never smoker	599	60.1%	537	59.3%	55	72.4%	
Body mass index	26.9	6.5	26.9	6.5	26.9	5.8	0.292
Disability							0.445
No walking device needed	626	63.0%	560	62.0%	53	70.7%	
Unilateral support (Cane)	127	12.8%	120	13.3%	6	8.0%	
Bilateral support (Walker)	104	10.4%	97	10.7%	7	9.3%	
Wheelchair	137	13.8%	127	14.1%	9	12.0%	
Diagnosis							
Relapsing‐remitting MS	574	57.4%					
Secondary progressive MS	215	21.5%					
Primary progressive MS	118	11.8%					
NMOSD	77	7.7%					
Other	16	1.6%					
Disease duration (Year, SD)	8.5	8.3	9.1	8.3	3.0	6.0	**<0.001**
Last DMT used							**<0.001**
Natalizumab	278	27.8%	276	30.4%	2	2.6%	
None	163	16.3%	115	12.7%	38	49.4%	
Dimethyl fumarate	153	15.3%	151	16.6%	1	1.3%	
Fingolimod	155	15.5%	155	17.1%	0	0.0%	
Glatiramer acetate	100	10.0%	97	10.7%	2	2.6%	
Interferon	66	6.6%	62	6.8%	3	3.9%	
Teriflunomide	15	1.5%	15	1.7%	0	0.0%	
Azathioprine	15	1.5%	0	0.0%	15	19.5%	
Mycophenolate mofetil	10	1.0%	1	0.1%	7	9.1%	
Other/missing	47	4.7%	35	3.9%	9	11.7%	
Time since last DMT (months)	7.2	20.0	7.8	19.5	4.5	8.0	**0.037**

Bold *P*‐values indicate *P*> 0.05 and are considered statistically significant.

*N*, number; SD, standard deviation; MS, multiple sclerosis; NMOSD, neuromyelitis optica spectrum disorder; DMT, disease‐modifying therapy.

^1^ Comparing baseline characteristics of MS and NMOSD patients.

**Table 2:** Percentage of patients who experienced a serious safety event and incidence rate per 1000 person‐years of rituximab/ocrelizumab treatment.

	Total *N* (%)	Incidence rate per 1000 person‐years (95% CI)	By diagnosis		
			MS *N* (%)	NMOSD *N* (%)	*P*‐value^2^
Number of infections resulting in:	100 (8.6%)^1^	38.6 (31.5, 46.7)	85 (7.7%)	10 (13.0%)	0.103
Hospitalization	79 (6.5%)^1^	30.5 (24.3, 37.8)	71 (6.3%)	8 (10.4%)	0.155
IV antibiotics (without hospitalization)	6 (0.6%)	2.3 (0.9, 4.8)	6 (0.7%)	0 (0.0%)	1.000
Extended dosing antibiotics	15 (1.5%)	5.8 (3.4, 9.3)	12 (1.3%)	3 (3.9%)	0.106
Infusion reaction requiring hospitalization	0 (0.0%)	0.0 (.)	0.0 (.)	0.0 (.)	
Malignant Cancer	9 (0.9%)	3.5 (1.7, 6.4)	9 (1.0%)	0 (0.0%)	1.000
New autoimmune disease diagnosis	6 (0.6%)	2.3 (0.9, 4.8)	4 (0.4%)	2 (2.6%)	0.074
Thromboembolic event (Non‐superficial)	8 (0.8%)	3.1 (1.4, 5.9)	6 (0.7%)	2 (2.6%)	0.124
Lymphopenia					
<500 cells/mm^3^	48 (5.1%)	19.2 (14.3, 25.2)	38 (4.5%)	10 (13.9%)	**0.003**
<200 cells/mm^3^	2 (0.2%)	0.8 (0.1, 2.6)	2 (0.2%)	0 (0.0%)	1.000
Neutropenia					
<1000 cells/mm^3^	14 (1.5%)	5.6 (3.2, 9.2)	13 (1.5%)	1 (1.4%)	1.000
<500 cells/mm^3^	11 (1.2%)	4.4 (2.3, 7.7)	11 (1.3%)	0 (0.0%)	1.000
Low IgG values					
<500 mg/dL	38 (5.2%)	17.8 (12.8, 24.2)	25 (3.7%)	13 (24.1%)	**<0.001**
<300 mg/dL	8 (1.1%)	3.7 (1.7, 7.1)	1 (0.2%)	7 (13.0%)	**<0.001**
Mortality	14 (1.4%)	5.4 (3.1, 8.8)	12 (1.3%)	2 (2.6%)	0.301

Bold *P*‐values indicate *P*> 0.05 and are considered statistically significant.

*N*, number of events occurred; %, percent of patients who experienced event; MS, Multiple Sclerosis; NMOSD, Neuromyelitis Optica Spectrum Disorder

^1^ Sixty‐five patients (57 MS; 8 NMO) were hospitalized a total of 79 times (71 MS; 8 NMO). Nine patients (9 MS; 0 NMO) were hospitalized multiple times due to infections.

^2^ Comparing serious safety events of MS and NMOSD patients

### Hematological abnormalities

**Figure 2:**
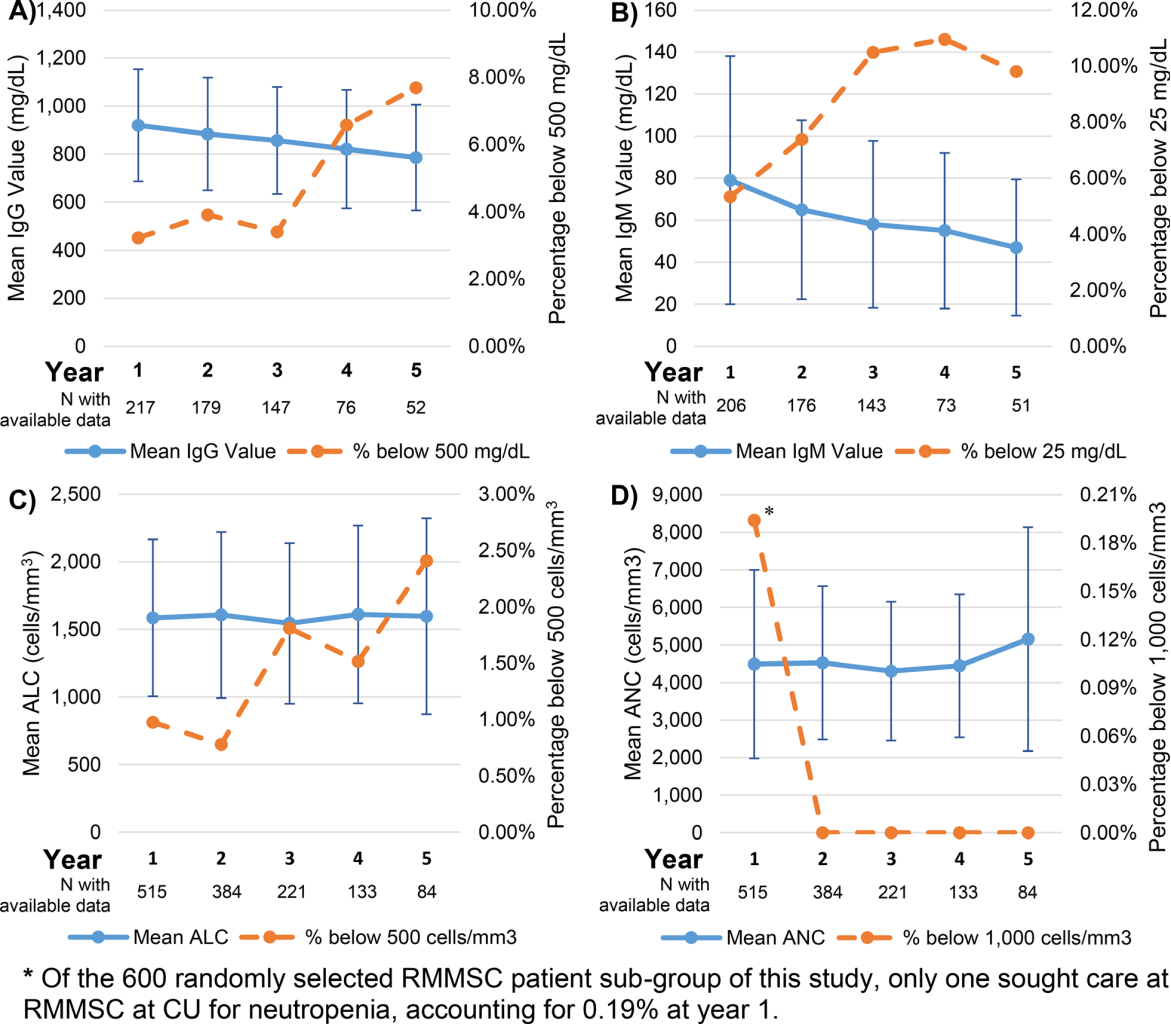
(A) Mean IgG value with standard deviation and percentage of patients with an IgG value <500 mg/dL by year of rituximab treatment. (B) Mean IgM value with standard deviation and percentage of patients with an IgM value <25 mg/dL by year of rituximab treatment. (C) Mean absolute lymphocyte count (ALC) with standard deviation and percentage of patients with an ALC <500 cells/mm^3^ by year of rituximab treatment. (D) Mean absolute neutrophil count (ANC) with standard deviation and percentage of patients with an ANC <500 cells/mm^3^ year of rituximab treatment.

The mean time to lymphopenia is 17.8 (SD:20.6) months with cases ranging from <1 month to 98 months after initial dose (median:11). The mean time to neutropenia is 17.9 (SD:13.3) months and range from <1 to 41 months (median:15). Of the 14 who experienced transient neutropenia, three experienced neutropenic fever, including two with unknown probable viral infections and one with suspected mononucleosis. Despite neutropenia, eight patients were re‐challenged with rituximab/ocrelizumab with no neutropenia reoccurrence. Patients who experienced neutropenia and those who did not have a mean BMI of 23.7 and 26.8, respectively (*P* = 0.079). The mean time to hypogammaglobulinemia was 44.8 (SD:20.6) months, median is 42 months, range from < 1 to 137 months. Figure 2 demonstrates the mean IgG value, mean IgM value, mean ALC, and mean ANC by year of treatment for 600 randomly selected rituximab‐treated patients from RMMSC at CU patients.

### Serious infections

Of the 1000 patients included, 65 (6.5%) were hospitalized for infection and nine patients were hospitalized multiple times. At the time of first hospitalization, patients had a mean ALC of 1427 cells/mm^3^ (*n* = 65), mean ANC of 6634 cells/mm^3^ (*n* = 65), and mean IgG of 716 mg/dL (*n* = 33). Five (15.5%) hospitalized patients had IgG values less than 500 mg/dL, of which three (9.1%) were below 300 mg/dL. Table S5 lists the types of infections experienced, with urinary tract infections and sepsis being the most common. At the time of serious infection, patients had received a mean cumulative dose of 3705 mg rituximab (median 2500 mg). Two of these patients had switched to ocrelizumab, each having received 600 mg prior to infection. The mean time since first and last infusion to infection is 25.1 and 3.5 months, respectively.

### Risk factors for serious infection

In a multivariable model (stepwise selection), there was a dramatic increase in the risk of first infection with increasing ambulatory disability (non‐ambulatory vs. fully ambulatory patients OR: 8.56; 95%CI: 4.47–16.39; Table 3). Additionally, increased risk was demonstrated in patients with the history of prior immunosuppression (OR: 2.41; 95%CI: 1.19–4.86) and males (OR: 2.16; 95%CI: 1.24–3.77). Total time on rituximab/ocrelizumab modestly increases risk per year (OR: 1.33; 95%CI: 1.17–1.51).

**Table 3:** Bivariate and multivariable regression analyses assessing risk factors for experiencing first infection resulting in hospitalization, IV antibiotics or extended dosing antibiotics during rituximab/ocrelizumab treatment

	Bivariate analysis		Multivariable analysis (All variables included)			Multivariable analysis (stepwise selection)	
	Odds ratio	*P*‐value	Odds ratio	*P*‐value		Odds ratio	*P‐*value
Age (per year)	**1.02** (1.00, 1.04)	**0.015**	1.00 (0.97, 1.03)	0.986			N.S.
Gender		**0.003**		**0.011**			**0.007**
Female	*Reference*		*Reference*			*Reference*	
Male	**2.01** (1.27, 3.19)		**2.15** (1.20, 3.85)			**2.16** (1.24, 3.77)	
Race		0.639		0.388			N.S.
White	*Reference*		*Reference*				
Black	1.01 (0.53, 1.94)		0.91 (0.38, 2.19)				
Other	0.68 (0.30, 1.53)		2.28 (0.67, 7.70)				
Ethnicity		0.310		0.195			N.S.
Non‐hispanic	*Reference*		*Reference*				
Hispanic	0.64 (0.27, 1.51)		0.38 (0.09, 1.65)				
Smoking status		0.878		0.637			N.S.
Never smoker	*Reference*		*Reference*				
Former smoker	1.14 (0.68, 1.92)		1.28 (0.68, 2.44)				
Current smoker	1.10 (0.56, 2.19)		1.38 (0.60, 3.20)				
Body mass index	**0.96** (0.92, 1.00)	**0.033**	0.98 (0.94, 1.03)	0.507			
Disability		**<0.001**		**<0.001**			**<0.001**
No walking device needed	*Reference*		*Reference*			*Reference*	
Unilateral support (Cane)	**1.91** (0.87, 4.22)		**1.58** (0.60, 4.12)			**1.69** (0.70, 4.09)	
Bilateral support (Walker)	**3.27** (1.58, 6.77)		**2.85** (1.11, 7.33)			**3.14** (1.34, 7.37)	
Wheelchair	**8.61** (4.92, 15.1)		**6.84** (3.06, 15.30)			**8.56** (4.47, 16.39)	
Diagnosis		**<0.001**		0.711			N.S.
Relapsing‐remitting MS	*Reference*		*Reference*				
Progressive MS	**4.20** (2.49, 7.10)		1.23 (0.58, 2.64)				
NMOSD	**3.74** (1.70, 8.23)		1.62 (0.46, 5.69)				
Disease duration (per year)	**1.03** (1.01, 1.06)	**0.008**	1.02 (0.99, 1.06)	0.255			N.S.
Immunosuppression/chemotherapy history		**<0.001**		0.136			**0.039**
No history	*Reference*		*Reference*			*Reference*	
Prior history	**3.08** (1.77, 5.37)		2.11 (1.00, 4.41)			**2.41** (1.19, 4.86)	
Current history	**1.04** (0.24, 4.51)		1.74 (0.28, 10.98)			**2.03** (0.41, 10.12)	
Induction dose (per gram)	**2.29** (1.46, 3.60)	**<0.001**	1.41 (0.79, 2.52)	0.249			N.S.
Time on rituximab (per year)	**1.29** (1.17, 1.42)	**<0.001**	**1.31** (1.13, 1.51)	**<0.001**		**1.33** (1.17, 1.51)	**<0.001**

Bold *P‐*values indicate *P*> 0.05 and are considered statistically significant. “Bivariate” indicates two variables were included in the analysis, including 1) the outcome of the first infection and 2) the risk factor in the first column of the corresponding row.

N.S, Not significant; MS, multiple sclerosis; NMOSD, neuromyelitis optica spectrum disorder.

Patients with IgG values < 500 mg/dL at any time had increased odds of infection resulting in hospitalization, extended dosing antibiotics or IV antibiotics before and after adjustment (adjusted OR: 3.15; 95%CI: 1.16–8.55) (Table S6). Patients with lymphopenia at any time also had increased odds of infection (adjusted OR: 2.55; 95%CI: 1.12–5.81) (Table S7).

### Infusion reactions

No patients had an infusion reaction considered life‐threatening or resulting in hospitalization (grade 3). One patient received epinephrine in the Emergency Department post‐infusion but was able to continue rituximab on a slower infusion schedule.

### Malignant cancer, new autoimmune disease, and serious thromboembolic events

Nine (0.9%) patients were diagnosed with cancer with a mean time from rituximab start to diagnosis of 3.2 years (four breast cancers, two papillary thyroid cancers, one glioblastoma, one bladder cancer, and one metastatic squamous cell carcinoma). Six (0.6%) patients were diagnosed with a new autoimmune disease with a mean time from rituximab start to diagnosis of 1.6 years (one Crohn Disease, one Celiac Disease, one Scalp Psoriasis, one Type 1 Diabetes, one Autoimmune thyroiditis, and one Alopecia). Eight (0.8%) patients were diagnosed with a thromboembolic event with a mean time from rituximab start to diagnosis of 2.6 years (five deep vein thrombosis, one pulmonary embolism, two deep vein thrombosis and pulmonary embolisms).

### Mortality

Fourteen (1.4%) patients died within 12 months of their last rituximab dose. Three patients committed suicide 15, 43, and 46 days after their last infusion, having received 1, 3, and 11 total doses of rituximab. Additional causes of deaths were glioblastoma (1), pneumonia and sepsis (1), and pulmonary embolism (1). Causes of eight deaths were unverified but were attributed to severe neurologic diseases by treating clinician: seven were wheelchair‐bound at last follow‐up, and one was diagnosed with malignant NMOSD with the quick progression of disease.

### Subgroup analyses

Baseline and follow‐up characteristics were assessed by the study center (Tables S2–S4), among those who experienced an SSE compared to those who did not (Tables S8–S9) and by SSE type (Table S10). Outcomes were assessed by age, disability, and immunosuppression/chemotherapy history (Tables S11–S14). Patients ≥55 years of age had a greater proportion who were hospitalized for an infection, experienced a thromboembolic event, or had lymphopenia than those <55 years of age. Wheelchair‐bound patients had a greater proportion who were hospitalized for an infection, experienced a thromboembolic event, or died compared to those with no assistive device, unilateral support, or bilateral support.

## Discussion

Our real‐world study investigated the safety of long‐term rituximab for MS, NMOSD, and related neurologic disorders at two large academic MS centers with a heterogeneous, ethnically diverse sample size of 1000 patients. Rituximab had an overall favorable safety profile in the MS and NMOSD population. The most common SSEs experienced were infections. The overall rate of infections resulting in hospitalization, IV antibiotics, and extended dosing antibiotics was 38.6 per 1000 PY of rituximab treatment, which is nearly identical to the rate of 39.4/1000 PY reported in a long‐term study of rituximab‐treated RA patients.^10^ Importantly, types of infections resulting in hospitalization were similar to what is seen in the general MS and NMOSD populations with the exception of hepatitis B reactivation and pulmonary aspergillus. No cases of PML were observed. However, our results are discordant with two large Swedish studies. One documented a lower prevalence of high‐grade infections (1.7%) in rituximab‐treated MS patients.^8^ A second study demonstrated a lower incidence rate of 19.7 per 1000 PY.^16^ The lower prevalence and incidence rate may be due to their shorter mean follow‐up time (21.8 and 24.0 months vs. 31.1 months in our study), the inclusion of more RRMS, potentially less disabled patients, and different methodologies of extracting SSE data.^8^, ^16^ Another methodological difference is that we included in our follow‐up patients who were transitioned from rituximab to ocrelizumab. This decision was made because the mechanism of action, efficacy, and safety profile of ocrelizumab in MS are very similar to rituximab. Analysis of infection rates in the ‘rituximab only group’ and ‘rituximab‐to‐ocrelizumab group’ did not show significant differences.

We observed a dramatic increase in infection risk for patients with increasing levels of ambulatory disability. Similarly, previous findings attributed 91.8% of hospitalizations due to infections to be likely related to advanced MS.^17^ These observations have important implications for the proper selection of patients for rituximab and likely ocrelizumab therapy. Given the difference in infection rates among different disability strata, it is extremely important – perhaps even imperative – that pharmaceutical companies stratify infection rates based on patient disability in clinical trials. Importantly, the benefits of anti‐CD20 treatment in MS are much higher in the younger, less disabled patients early in the disease as compared to more disabled patients who have entered a progressive phase. Thus, risk:benefit ratio increases with disease duration and needs to be continually reassessed.

Hypogammaglobulinemia also significantly increased odds of infection, though the majority of patients with low IgG values did not have a serious infection. This finding is in line with a previous study of rituximab in RA, which found that patients with low IgG had higher rates of serious infection.^10^ Our study confirms no association between low IgM and infections, a result also demonstrated by the RA study.^10^ IgM levels tend to decrease in treated patients earlier than IgG levels (Fig. 2). This raises the question of whether low IgM values are predictive of future low IgG values, which we intend to investigate in a future study. Effect of low IgG values <500 mg/dL on the odds of infection was more pronounced than that of lymphopenia (<500 cells/mm^3^) (Tables S6 and S7). However, it is possible that these associations may be attenuated, as some clinicians may alter or stop rituximab/ocrelizumab treatment based on critically low lab values prior to an infection occurring. Monitoring IgG levels and lymphocyte counts in combination with clinical risk factors may allow clinicians to customize treatment and decrease the risk of serious infections.

Regardless of infections, hypogammaglobulinemia was the most common laboratory abnormality observed. IgG values decreased over time, suggestive of a cumulative dose‐dependent response. This observation is consistent with studies of lymphoma and RA populations, which demonstrated a higher dosage or having >1 rituximab infusion to be associated with increased risk of hypogammaglobulinemia.^18^, ^19^, ^20^ Additionally, prior immunosuppression/chemotherapy exposure and diagnosis of NMOSD also correlate with lower IgG values.

In our study, the majority of neutropenia cases occurred within the first two years, but clinicians should continue to be vigilant for the potential of late‐onset neutropenia. Importantly, as 8 of the 14 with transient neutropenia were re‐challenged with rituximab/ocrelizumab with no neutropenia reoccurrence, continued treatment with rituximab/ocrelizumab appeared safe. Lymphopenia occurred more commonly in older patients and those with NMOSD, independently in our study. Previous research in MS and NMOSD patients demonstrated a marked decrease in lymphocytes after the first rituximab infusion, although subsequent doses did not result in a significant drop.^21^ However, this study only investigated up to three doses of rituximab, and lymphocyte decreases may occur with long‐term treatment as suggested by our results. In addition, NMOSD patients had greater follow‐up time and a mean cumulative anti‐CD20 dose twice that of MS patients, which likely contributed to the increase of lymphopenia and low IgG values seen in these patients. Future studies should employ adjusted analyses aimed to further explore the potential differences between diagnoses.

The mortality rate in rituximab‐treated patients was 5.4 per 1000 PY. Most of these patients were severely disabled with end‐stage disease. Nevertheless, in eight cases, the cause of death could not be verified, and one patient death was caused by pneumonia and sepsis, cases in which we cannot rule out rituximab as a contributing factor. Additionally, three suicides occurred resulting in an incidence rate of 1.2/1000 PY, although increased rates of suicide have been associated with an MS diagnosis.^22^

Infusion reactions are commonly cited AEs, particularly with the first infusion, however, they are rarely serious as seen in our study with no infusion deemed life‐threatening or resulting in hospitalization.^3^, ^4^, ^8^ The low rate could be due to our use of high‐dose steroids and anti‐histamines as part of standard pretreatment protocol and careful attention to titration rates by experienced infusion personnel.

The rate of malignant cancer in our study was similar to the general population (3.5/1000 PY in general population vs. 4.2/1000 PY).^23^ This is consistent with a nationwide register‐based cohort study conducted in Sweden, which found no difference in the risk of invasive cancer in rituximab‐treated MS patients compared to the general population.^24^ MS and NMOSD are associated with an increased risk of non‐superficial thromboembolic events.^25^, ^26^ In our study, rates of thromboembolism were higher (0.8%) than previously reported citing a rate 1.2/1000 PY, this was likely due to inclusion of older and potentially more disabled patients, known risk factors for thromboembolic events.^25^, ^26^, ^27^ Our results suggest the observed increase in new autoimmune diseases appeared to be driven by NMOSD (Table 2), as NMOSD is associated with other immune‐mediated diseases.^28^

Our large case series study was conducted at two large academic centers, possibly limiting generalizability. Other limitations include its retrospective design (with potential bias from loss to follow‐up) and the constraint of reviewing only electronic medical records available at the two centers. If a patient sought care for an SSE at an outside hospital and did not report it during a subsequent routine clinical encounter with the participating center, the event would not have been captured by our study design. Furthermore, patients may not accurately remember the details of the SSE, such as the type of infection. While these limitations may result in the number of events reported by our study to be lower than those truly experienced, trends could still be readily assessed. Moreover, the selected SSEs were routinely assessed by our clinicians through oral accounts during clinic visits or phone encounters and would be regularly captured through documentation practices and our rigorous review of all notes. Although unconfirmed self‐reported outcomes were included, data were collected at the closest available time point to the event of interest to reduce inaccuracies due to recall. Furthermore, we were unable to collect and account for comorbidities, which may increase the risk for infection for some patients. Additionally, as rituximab is used off‐label, there is no consensus on dosing schedules. In recent years, standard practices at RMMSC at CU and NYUMSCC typically prescribe an induction dose of 1000 mg, followed by a maintenance dosing of 500 or 1000 mg every 6 months depending on physician preference, however, previously an induction dose of 2000 mg was used. Finally, our study lacks a comparator group limiting our ability to provide direct evidence or interpret events to be drug‐related. However, our findings are consistent with previous literature.

The strengths of our study include two‐center design, large sample size, racially diverse population, relatively long duration of follow‐up, and careful review of all available records for SSEs. Our study documented relatively low rates of SSEs in a large group of rituximab‐treated patients with inflammatory disorders of the central nervous system. Infections were the most common SSEs, there was only one opportunistic infection, one hepatitis B reactivation, and no cases of PML. We identified significant risk factors for infections, which may lead to a better selection of appropriate patients for therapy. High efficacy of rituximab in phase 2 and observational studies^3^, ^4^, ^5^ combined with observed low SSE rates among non‐disabled patients with relapsing MS suggests that B‐cell depleting therapy may be appropriate in early disease even as a first‐line treatment and may reduce disability‐related serious safety events in the long run. Through assessing risk factors and continual monitoring of IgG values and lymphocyte counts, clinicians may reduce risk of SSEs during rituximab treatment in MS, NMOSD, and related disorders.

## Conflicts of Interest

Brandi Vollmer MPH has nothing to disclose. Asya I. Wallach MD received fellowship support from the National MS Society and Biogen, and has consulted for Biogen. John R Corboy MD has received grant support from Novartis, Med Day, NMSS, and PCORI; sits on a steering committee for a clinical trial with Novartis; consults with Mylan on a legal issue; receives honorarium for speaking from the Rocky Mountain MS Center and PRIME CME, and receives compensation as editor of Neurology Clinical Practice. Karolina Dubovskaya has nothing to disclose. Enrique Alvarez MD has consulted for Actelion/Janssen, Bayer, Biogen, Celgene, EMD Serono, Genentech, Genzyme, Novartis, and TG Therapeutics; and received research funding from Biogen, Genentech, Novartis, TG Therapeutics, and Rocky Mountain MS Center. Ilya Kister MD has served on the scientific advisory board of Biogen and Genentech; consulted for Biogen Idec and Genentech/Roche; received research support from Biogen Idec, Serono, Novartis, Genzyme, Genentech, Guthy‐Jackson Charitable Foundation, NMSS.

## Supporting information


**Table S1**. Follow‐up characteristics for MS and NMOSD patients.
**Table S2**. Baseline characteristics by study center.
**Table S3**. Percentage of patients who experienced a serious safety event on rituximab/ocrelizumab by study center.
**Table S4**. Follow‐up characteristics by study center.
**Table S5**. Number of infections resulting in hospitalizations, IV antibiotics (without hospitalization) and extended dosing antibiotics.
**Table S6**. Descriptive statistics and odds ratio for patients experiencing first infection who had an IgG value < 500 mg/dL at any time compared to those with IgG values always ≥ 500 mg/dL.
**Table S7**. Descriptive statistics and odds ratio for patients experiencing first infection who had lymphopenia at any time compared to those without.
**Table S8**. Baseline characteristics for those who experience an SSE and those who do not.
**Table S9**. Follow‐up characteristics for those who experience an SSE and those who do not.
**Table S10**. Follow‐up characteristics for those who experience an SSE by type at time of SSE.
**Table S11**. Percentage of patients who experienced a serious safety event while on rituximab/ocrelizumab by age.
**Table S12**. Baseline characteristics by disability.
**Table S13**. Percentage of patients who experienced a serious safety event while on rituximab/ocrelizumab by disability at baseline.
**Table S14**. Percentage of patients who experienced a serious safety event on rituximab/ocrelizumab by immunosuppression/chemotherapy history.
