# Supplementary material for: Serious safety events in rituximab‐treated multiple sclerosis and related disorders
Source: Ann Clin Transl Neurol. 2020 Aug 6;7(9):1477–87. doi: 10.1002/acn3.51136 (PMC7480911; doi:10.1002/acn3.51136)
Supplement: Supplementary file 1 — Table S1. Follow‐up characteristics for MS and NMOSD patients. Table S2. Baseline characteristics by study center. Table S3. Percentage of patients who experienced a serious safety event on rituximab/ocrelizumab by study center. Table S4. Follow‐up characteristics by study center. Table S5. Number of infections resulting in hospitalizations, IV antibiotics (without hospitalization) and extended dosing antibiotics. Table S6. Descriptive statistics and odds ratio for patients experiencing first infection who had an IgG value < 500 mg/dL at any time compared to those with IgG values always ≥ 500 mg/dL. Table S7. Descriptive statistics and odds ratio for patients experiencing first infection who had lymphopenia at any time compared to those without. Table S8. Baseline characteristics for those who experience an SSE and those who do not. Table S9. Follow‐up characteristics for those who experience an SSE and those who do not. Table S10. Follow‐up characteristics for those who experience an SSE by type at time of SSE. Table S11. Percentage of patients who experienced a serious safety event while on rituximab/ocrelizumab by age. Table S12. Baseline characteristics by disability. Table S13. Percentage of patients who experienced a serious safety event while on rituximab/ocrelizumab by disability at baseline. Table S14. Percentage of patients who experienced a serious safety event on rituximab/ocrelizumab by immunosuppression/chemotherapy history. [file ACN3-7-1477-s001.pdf]

Supplemental Data

Follow-Up Characteristics by Diagnosis.

Table S1. Follow-up characteristics for MS and NMOSD patients.

	**MS** N=907		**NMOSD** N=77		
**Follow Up Characteristics**	**N or Mean**	**SD or %**	**N or Mean**	**SD or %**	**p-value**[α]
**Cumulative rituximab Dose (mg)**	3,713 (median=3,000)	3015	7,526 (median=6,000)	6,272	**<0.001**
**Patients who switched to ocrelizumab**	169	18.9%	0	0.0%	-
**Cumulative ocrelizumab dose (mg)***	1,141 (median= 1,200)	414	-	-	-
**Time on rituximab/ocrelizumab (months)**	29.7	21.0	47.2	40.0	**<0.001**

Bold p-values indicate p>0.05 and are considered statistically significant.
*Calculated of those who switched to ocrelizumab
[α]Comparing MS and NMOSD patients

Baseline Characteristics by Study Center

Table S2. Baseline characteristics by study center.

	**RMMSC** N=725		**NYUMSCC** N=275		
**Baseline Characteristics**	**N or Mean**	**SD or %**	**N or Mean**	**SD or %**	**p-value**[α]
**Age (Years, SD)**	44.3	12.4	39.3	12.5	**<0.001**
**Gender – Female**	496	68.4%	191	69.5%	0.751
**Race**					**<0.001**
White	560	77.2%	118	42.9%	
Black	57	7.9%	82	29.8%	
Other	57	7.9%	60	21.8%	
Unknown	51	7.0%	15	5.5%	
**Ethnicity**					**<0.001**
Hispanic	64	8.8%	43	15.6%	
Non-Hispanic	604	83.3%	185	67.3%	
Unknown	57	7.9%	47	17.1%	
**Smoking Status**					**0.029**
Current Smoker	106	14.6%	25	9.2%	
Former Smoker	199	27.5%	67	24.7%	
Never Smoker	420	57.9%	179	66.1%	
**Body Mass Index**	26.8	6.5	26.9	6.4	0.928
**Disability**					**0.019**
No walking device needed	438	60.4%	188	69.9%	
Unilateral support (Cane)	105	14.5%	22	8.2%	
Bilateral support (Walker)	77	10.6%	27	10.4%	
Wheelchair	105	14.5%	32	11.9%	
**Diagnosis**					**<0.001**
Relapsing-Remitting MS	426	58.8%	147	53.5%	
Secondary Progressive MS	176	24.3%	39	14.2%	
Primary Progressive MS	98	13.5%	21	7.6%	
NMOSD	23	3.2%	54	19.6%	
Other	2	0.3%	14	5.1%	
**Disease Duration**	9.2	8.7	6.6	6.5	**<0.001**
**Last DMT Used**					**<0.001**
Azathioprine	2	0.3%	13	4.7%	
Dimethyl fumarate	106	14.6%	47	17.1%	
Fingolimod	127	17.5%	28	10.2%	
Glatiramer Acetate	82	11.3%	18	6.5%	
Interferon	56	7.7%	10	3.6%	
Mycophenolate mofetil	0	0.0%	10	3.6%	
Natalizumab	210	29.0%	68	24.7%	
None	102	14.1%	61	22.2%	
Other/Missing	29	0.4%	16	5.8%	
Teriflunomide	11	1.5%	4	1.5%	
**Time Since Last DMT (Months)**	7.8	19.8	5.5	20.6	0.171

Bold p-values indicate p>0.05 and are considered statistically significant. RMMSC: Rocky Mountain Multiple Sclerosis Clinic; NYUMSCC: New York University Multiple Sclerosis Care Center; N: number; SD: standard deviation; MS: multiple sclerosis; NMOSD: neuromyelitis optica spectrum disorder; DMT: disease modifying therapy.

[α]Comparing RMMSC and NYUMSCC patients

Table S3. Percentage of patients who experienced a serious safety event on rituximab/ocrelizumab by study center.

	**Total** N (%)	**Diagnosis**		**p-value**[α]
		**RMMSC** N (%)	**NYUMSCC** N (%)	
**Infections resulting in:**	100 (8.6%)*	85 (9.7%)	15 (5.5%)	**0.019**
Hospitalization	79 (6.5%)*	70 (7.7%)	9 (3.3%)	**0.012**
IV antibiotics (without hospitalization)	6 (0.6%)	6 (0.8%)	0 (0.0%)	0.196
Extended dosing antibiotics	15 (1.5%)	9 (1.2%)	6 (2.2%)	0.260
**Infusion reaction requiring hospitalization**	0 (0.0%)	0 (0.0%)	0 (0.0%)	
**Malignant Cancer**	9 (0.9%)	8 (1.1%)	1 (0.4%)	0.458
**New autoimmune disease diagnosis**	6 (0.6%)	3 (0.4%)	3 (1.1%)	0.355
**Thromboembolic event (Non-superficial)**	8 (0.8%)	6 (0.8%)	2 (0.7%)	0.372
**Lymphopenia**				
<500 cells/mm3	48 (5.1%)	29 (4.3%)	19 (7.4%)	0.056
<200 cells/mm3	2 (0.2%)	1 (0.1%)	1 (0.4%)	0.476
**Neutropenia**				
<1000 cells/mm3	14 (1.5%)	9 (1.3%)	5 (2.0%)	0.483
<500 cells/mm3	11 (1.2%)	8 (1.2%)	3 (1.2%)	1.000
**Low IgG Values**				
<500 mg/dL	38 (5.2%)	30 (5.4%)	8 (4.3%)	0.545
<300 mg/dL	8 (1.1%)	3 (0.5%)	5 (2.6%)	**0.023**
**Mortality**	14 (1.4%)	12 (1.7%)	2 (0.7%)	0.372

Bold p-values indicate p>0.05 and are considered statistically significant. N: number of events occurred; %: percent of patients who experienced event; RMMSC: Rocky Mountain Multiple Sclerosis Center; NYUMSCC: New York University Multiple Sclerosis Care Center

* Sixty-five patients were hospitalized a total of 79 times. Nine patients were hospitalized multiple times due to infections.

[α]Comparing RMMSC and NYUMSCC patients

Table S4. Follow-up characteristics by study center.

	RMMSC N=725		NYUMSCC N=275		
**Follow-Up Characteristics**	**N or Mean**	**SD or %**	**N or Mean**	**SD or %**	**p-value**[α]
**Cumulative rituximab dose (mg)**	3,152 (median=2,500)	2,261	6,410 (median=5,000)	4,984	**<0.001**
**Patients who switched to ocrelizumab**	131	18.1%	38	13.8%	0.109
**Cumulative ocrelizumab dose (mg)***	1,191 (median= 1,200)	413	971 (median= 1,200)	378	**0.004**
**Cumulative anti-CD20 dose**	3,368 (median= 2,700)	2,287	6,551 (median= 5,000)	4,947	**<0.001**
**Time on rituximab/ocrelizumab (months)**	28.7 (median=23)	21.5	37.6 (median=29)	27.1	**<0.001**

Bold p-values indicate p>0.05 and are considered statistically significant. RMMSC: Rocky Mountain Multiple Sclerosis Clinic; NYUMSCC: New York University Multiple Sclerosis Care Center

*Calculated of those who switched to ocrelizumab

[α]Comparing RMMSC and NYUMSCC patients

Types of Infections

Table S5. Number of infections resulting in hospitalizations, IV antibiotics (without hospitalization) and extended dosing antibiotics.

Infection	**Hospitalization*** n=65 (6.5% of patients)	**IV Antibiotics (without Hospitalization)** n=6 (0.6% of patients)	**Extended Dosing Antibiotics** n=15 (1.5% of patients)
	**N**	**N**	**N**
**Urinary Tract Infection**	35	2	10
**Sepsis**	22	-	-
**Pneumonia**	20	-	1
**Cellulitis**	5	1	-
**Abscess**	3	-	-
**C. Diff. Colitis**	2	-	-
**Kidney infection**	2	-	-
**Appendicitis**	2	-	-
**Influenza**	1	-	-
**Respiratory syncytial virus**	1	-	-
**Sinusitis / Bronchitis**	1	1	-
**Non-specific fever**	1	-	--
**Wound infection**	2	1	-
**Stomach infection**	1	-	-
**Bacteremia**	1	-	1
**Hep B reactivation**	1	--	-
**Pulmonary aspergillosis**	1	-	-
**Osteomyelitis**	1	-	1
**Viral pleural & pericardial effusion**	1	-	-
**Skin and joint infection**	-	1	1
**Bacterial Vaginosis**	-	-	1

*Some patients may have had multiple infections resulting in hospitalization

Association between Hypogammaglobulinemia and Infections

Table S6. Descriptive statistics and odds ratio for patients experiencing first infection who had an IgG value <500 mg/dL at any time compared to those with IgG values always ≥500 mg/dL

Infections resulting in Hospitalization Only[+]

	**Infections (Hospitalization)**	**No Infection**	**Total**
**IgG Value: <500 mg/dL**	8 (21.1%)	30 (78.9%)	38
**IgG Value: Always ≥500 mg/dL**	40 (5.7%)	660 (94.3%)	700

[+]Chi-square test **p-value: 0.002**

Logistic Regression Analysis

	**N**	**Odds Ratio** (95% CI)	**p-value**
**Unadjusted Logistic Regression**	738	**4.40** (1.89, 10.22)	**<0.001**
**Adjusted Logistic Regression***	646	**3.29** (1.13, 9.58)	**0.029**

Infections resulting in Hospitalization, Extended Dosing Antibiotics or IV Antibiotics^

	**All Infections**	**No Infection**	**Total**
**IgG Value: <500 mg/dL**	9 (23.7%)	29 (76.3%)	38
**IgG Value: Always ≥500 mg/dL**	52 (7.4%)	648 (92.6%)	700

^ Chi-square test **p-value: 0.004**

Logistic Regression Analysis

	**N**	**Odds Ratio** (95% CI)	**p-value**
**Unadjusted Logistic Regression**	738	**3.87** (1.74, 8.60)	**0.002**
**Adjusted Logistic Regression***	646	**3.15** (1.16, 8.55)	**0.024**

Note: Lab value<500 mg/dL at least once at any time, not necessarily at time of infection
Bold p-values indicate p>0.05 and are considered statistically significant.
*Adjusting for age at first infusion, gender, disease duration, diagnosis (Relapsing MS, Progressive MS or Other), and disability at baseline

Association between Lymphopenia and Infections

Table S7. Descriptive statistics and odds ratio for patients experiencing first infection who had lymphopenia at any time compared to those without.

Infections resulting in Hospitalization Only⁺

	Infections (Hospitalization)	No Infection	Total
**Lymphopenia <500 cells/mm^3**	8 (16.7%)	40 (83.3%)	48
**No Lymphopenia**	56 (6.3%)	832 (93.7%)	888

⁺Chi-square test **p-value: 0.013**

Logistic Regression Analysis			
	**N**	**Odds Ratio** (95% CI)	**p-value**
**Unadjusted Logistic Regression**	936	**2.97** (1.33, 6.65)	**0.008**
**Adjusted Logistic Regression***	926	**2.27** (0.93, 5.54)	**0.006**

Infections resulting in Hospitalization, Extended Dosing Antibiotics or IV antibiotics^

	All Infections	No Infection	Total
**Lymphopenia <500 cells/mm^3**	10 (20.8%)	38 (79.2%)	48
**No Lymphopenia**	69 (77.7%)	819 (92.2%)	888

^ Chi-square test **p-value: 0.005**

Logistic Regression Analysis			
	**N**	**Odds Ratio** (95% CI)	**p-value**
**Unadjusted Logistic Regression**	936	**3.12** (1.49, 6.53)	**0.003**
**Adjusted Logistic Regression***	926	**2.55** (1.12, 5.81)	**0.012**

Note: Lab value<500 mg/dL at least once at any time, not necessarily at time of infection
Bold p-values indicate p>0.05 and are considered statistically significant.
*Adjusting for age at first infusion, gender, disease duration, diagnosis (Relapsing MS, Progressive MS or Other), and disability at baseline

Characteristics of Patients who Experienced an SSE

Table S8. Baseline characteristics for those who experience an SSE and those who do not.

	**No SSE** N=823		**SSE*** N=177		
**Baseline Characteristics**	**N or Mean**	**SD or %**	**N or Mean**	**SD or %**	**p-value[ǂ]**
**Age (Years, SD)**	42.3	12.5	45.8	13.0	**0.001[β]**
**Gender – Female**	572	69.5%	115	65.0%	0.238[ǂ]
**Race**					0.616[ǂ]
White	553	67.2%	125	70.6%	
Black	116	14.1%	23	13.0%	
Other	96	11.7%	21	11.9%	
Unknown	58	7.1%	8	4.5%	
**Ethnicity**					0.267[ǂ]
Hispanic	90	10.9%	17	9.6%	
Non-Hispanic	642	78.0%	147	83.1%	
Unknown	91	11.1%	13	7.3%	
**Smoking Status**					0.515[ǂ]
Current Smoker	111	13.6%	20	11.3%	
Former Smoker	222	27.1%	44	24.9%	
Never Smoker	486	59.34%	113	63.8%	
**Body Mass Index**	27.1	6.6	25.8	6.0	**0.022[β]**
**Disability**					**<0.001[ǂ]**
No walking device needed	548	67.1%	78	44.1%	
Unilateral support (Cane)	103	12.6%	24	13.6%	
Bilateral support (Walker)	81	9.9%	23	13.0%	
Wheelchair	85	10.4%	52	29.4%	
**Diagnosis**					**<0.001[ǂ]**
Relapsing-Remitting MS	507	61.6%	66	37.3%	
Secondary Progressive MS	154	18.7%	61	34.5%	
Primary Progressive MS	101	12.3%	18	10.2%	
NMOSD	45	5.5%	32	18.1%	
Other	16	1.9%	0	0.0%	
**Disease Duration**	8.2	8.0	10.1	9.3	**0.006[β]**
**Last DMT Used**					0.102[ǂ]
Azathioprine	10	1.2%	5	2.8%	
Dimethyl fumarate	128	15.6%	25	14.1%	
Fingolimod	130	15.8%	25	14.1%	
Glatiramer Acetate	86	10.5%	14	7.9%	
Interferon	54	6.6%	5	7.9%	
Mycophenolate mofetil	5	0.6%	5	2.8%	
Natalizumab	220	26.7%	58	32.8%	
None	140	17.0%	23	13.0%	
Other/Missing	37	4.5%	8	4.5%	
Teriflunomide	13	1.6%	2	1.1%	
**Time Since Last DMT (Months, SD)**	7.7	20.6	5.0	17.0	0.141[β]

Bold p-values indicate p>0.05 and are considered statistically significant. SSE: serious safety event; N: number; SD: standard deviation; MS: multiple sclerosis; NMOSD: neuromyelitis optica spectrum disorder; DMT: disease modifying therapy.
*includes infusion reaction resulting in hospitalization, new diagnosis of malignant cancer, new diagnosis of an autoimmune disease, thromboembolic event (non-superficial), lymphopenia (<500 cells/mm^3), neutropenia (<1000 cells/mm^3), IgG values (<500 mg/dL), death or infections resulting in hospitalization, extended dosing antibiotics or IV antibiotics.

[α] Comparing those with no SSE to those with an SSE

[β] T-test

[ǂ] Chi-squared tests

Table S9. Follow-up characteristics for those who experience an SSE and those who do not.

	**No SSEs** N=823		**SSE^** N=177		
**Follow Up Characteristics**	**N or Mean**	**SD or %**	**N or Mean**	**SD or %**	**p-value** [α]
**Cumulative rituximab Dose (mg)**	3,749 (median=3,000)	3,262	5,215 (median=4,000)	4,309	**<0.001**[β]
**Patients who switched to ocrelizumab**	139	16.9%	30	17.0%	0.985[ᵻ]
**Cumulative ocrelizumab dose (mg)***	1,137 (median=1,200)	422	1,160 (median=1,200)	384	0.788[β]
**Time on rituximab/ocrelizumab (months)**	28.8 (median=24)	21.2	42.0 (median=32)	29.7	**<0.001**[β]

Bold p-values indicate p>0.05 and are considered statistically significant. SSE: serious safety event; N: number; SD: standard deviation.

^includes infusion reaction resulting in hospitalization, new diagnosis of malignant cancer, new diagnosis of an autoimmune disease, thromboembolic event (non-superficial), lymphopenia (<500 cells/mm^3), neutropenia (<1000 cells/mm^3), IgG values (<500 mg/dL), death or infections resulting in hospitalization, extended dosing antibiotics or IV antibiotics.

*Calculated of those who switched to ocrelizumab

[α] Comparing those with no SSE to those with an SSE

[β] T-test

[ᵻ] Chi-squared tests

Table S10. Follow-up characteristics for those who experience an SSE by type at time of SSE.

	**First Serious Infection** (N=86)	**Cancer** (N=9)	**Autoimmune disease** (N=6)	**Thromboembolic event** (N=8)
**Follow Up Characteristics**	**N or Mean [SD or %]**	**N or Mean [SD or %]**	**N or Mean [SD or %]**	**N or Mean [SD or %]**
**Cumulative rituximab Dose (mg)**	3,705 [3,346] Median=2,500	3,167 [2,511] Median=4,000	4,667 [4,761] Median=3,000	3,643 [2,116] Median=3,000
**Patients who switched to ocrelizumab**	2 (2.3%)	1 (11.1%)	0 (0.0%)	0 (0.0%)
**Cumulative ocrelizumab dose (mg)***	600 [.] Median=600	1,200 [.] Median=1,200	.	.
**Time on rituximab/ocrelizumab (months)**	25.1 [28.9] Median=19.0	38.8 (34.3) Median=50.0	19.0 (15.6) Median=16.5	31.5 [28.3] Median=27.0
	**Lymphopenia** (N=48)	**Neutropenia** (N=14)	**Low IgG Values** (N=38)	**Mortality** (N=14)
**Follow Up Characteristics**	**N or Mean [SD or %]**	**N or Mean [SD or %]**	**N or Mean [SD or %]**	**N or Mean [SD or %]**
**Cumulative rituximab Dose (mg)**	2,593 [2,408] Median=2,000	2,923 [1,801] Median=2,500	5,454 [4,812] Median=3,500	2,962 [1,738] Median= 2,500
**Patients who switched to ocrelizumab**	3 (6.3%)	1 (7.1%)	5 (13.2%)	0 (0.0%)
**Cumulative ocrelizumab dose (mg)***	500 [173] Median=600	300 [.] Median=300	1140 [537] Median = 1200	.
**Time on rituximab/ocrelizumab (months)**	17.8 [20.6] Median=11.0	17.9 [13.3] Median=15.0	44.8 [32.8] Median=42.0	30.4 [21.6] Median= 28.0

*Calculated of those who switched to ocrelizumab
SSE: serious safety event; N: number; SD: standard deviation.

Subgroup Analysis- By Age

Table S11. Percentage of patients who experienced a serious safety event while on rituximab/ocrelizumab by age.

	**Total** N (%)	**Age**		**p-value** [α]
		**<55 years N=808** N (%)	**≥55 years N=192** N (%)	
**Infections resulting in:**				
Hospitalization	79 (6.5%)*	53 (5.3%)*	26 (11.5%)*	**0.005**
IV antibiotics (without hospitalization)	6 (0.6%)	4 (0.5%)	2 (1.0%)	0.325
Extended dosing antibiotics	15 (1.5%)	12 (1.5%)	3 (1.6%)	1.000
**Infusion reaction requiring hospitalization**	0 (0.0%)	0 (0.0%)	0 (0.0%)	-
**Malignant Cancer**	9 (0.9%)	6 (0.7%)	3 (1.6%)	0.386
**New autoimmune disease diagnosis**	6 (0.6%)	5 (0.6%)	1 (0.5%)	1.000
**Thromboembolic event (Non-superficial)**	8 (0.8%)	4 (0.5%)	4 (2.1%)	**0.049**
**Lymphopenia**				
<500 cells/mm3	48 (5.1%)	29 (3.8%)	19 (10.7%)	**<0.001**
<200 cells/mm3	2 (0.2%)	2 (0.3%)	0 (0.0%)	0.656
**Neutropenia**				
<1000 cells/mm3	14 (1.5%)	11 (1.5%)	3 (1.7%)	0.736
<500 cells/mm3	11 (1.2%)	8 (1.1%)	3 (1.7%)	0.445
**Low IgG Values**				
<500 mg/dL	38 (5.2%)	28 (4.7%)	10 (7.0%)	0.256
<300 mg/dL	8 (1.1%)	5 (0.8%)	3 (2.1%)	0.186
**Mortality**	14 (1.4%)	10 (1.2%)	4 (2.1%)	0.323

Bold p-values indicate p>0.05 and are considered statistically significant. N: number of events occurred; %: percent of patients who experienced event; RMMSC: Rocky Mountain Multiple Sclerosis Center; NYUMSCC: New York University Multiple Sclerosis Care Center

* Sixty-five patients (43 <55 years; 22 ≥55 years) were hospitalized a total of 79 times (53 <55 years; 26 ≥55 years).

[α] Comparing those with <55 years to those ≥55 years

Nine patients (6 <55 years; 3 ≥55 years) were hospitalized multiple times due to infections.

Subgroup Analysis- By Disability

Table S12. Baseline characteristics by disability.

	**No Walking Device** N=626		**Unilateral Support** N=127		**Bilateral Support** N=104		**Wheelchair** N=137	
**Baseline Characteristics**	**N or Mean**	**SD or %**	**N or Mean**	**SD or %**	**N or Mean**	**SD or %**	**N or Mean**	**SD or %**
**Age (Years, SD)**	39.8	12.3	46.5	11.6	49.3	11.8	48.9	10.6
**Gender – Female**	439	70.1	77	60.6	78	75.0	88	64.2%
**Race**								
White	426	68.1	90	70.9	69	66.4	89	65.0
Black	73	11.7	25	19.7	17	16.4	23	16.8
Other	82	13.1	7	5.5	10	9.6	17	12.4
Unknown	45	7.2	5	3.9	8	7.8	8	5.8
**Ethnicity**								
Hispanic	72	11.5	12	9.5	10	9.6	11	8.0
Non-Hispanic	487	77.8	107	84.3	77	74.0	116	84.7
Unknown	67	10.7	8	6.3	17	16.4	10	7.3
**Smoking Status**								
Current Smoker	78	12.5	23	181.1	12	11.5	18	13.2
Former Smoker	156	24.9	39	30.7	31	29.8	40	29.4
Never Smoker	392	62.6	65	51.2	61	58.7	78	57.4
**Body Mass Index**	26.9	6.2	28.2	7.4	27.7	6.5	25.0	6.5
**Diagnosis**								
Relapsing-Remitting MS	480	76.7	51	40.2	24	23.1	17	12.4
Secondary Progressive MS	41	6.6	44	34.7	44	42.3	84	61.3
Primary Progressive MS	39	6.2	25	19.7	29	27.9	26	19.0
NMOSD	53	8.5	6	4.7	7	6.7	9	6.6
Other	13	2.1	1	0.8	0	0.0	1	0.7
**Disease Duration**	6.7	6.9	9.7	8.2	11.5	9.5	13.6	9.9
**Last DMT Used**								
Azathioprine	8	1.33	1	0.0	3	2.9	2	1.5
Dimethyl fumarate	103	16.5	16	12.6	21	20.2	12	8.8
Fingolimod	92	14.7	29	22.8	18	17.3	16	11.7
Glatiramer Acetate	63	10.1	11	8.7	5	4.8	21	15.3
Interferon	31	5.0	11	8.7	11	10.6	13	9.5
Mycophenolate mofetil	5	0.8	0	0.0	2	1.9	3	2.2
Natalizumab	161	58.1	35	12.6	31	11.2	50	18.1
None	130	20.8	15	11.8	6	5.8	11	8.0
Other/Missing	27	4.3	6	4.7	5	4.8	6	4.4
Teriflunomide	6	1.0	4	3.2	2	1.9	3	2.2
**Time Since Last DMT (Months)**	6.2	16.0	8.2	18.1	5.2	9.0	12.8	35.0

RMMSC: Rocky Mountain Multiple Sclerosis Clinic; NYUMSCC: New York University Multiple Sclerosis Care Center; N: number; SD: standard deviation; MS: multiple sclerosis; NMOSD: neuromyelitis optica spectrum disorder; DMT: disease modifying therapy.

Table S13. Percentage of patients who experienced a serious safety event while on rituximab/ocrelizumab by disability at baseline.

		Disability				
	**Total** N (%)	**No Device N= 626** N (%)	**Unilateral Support N=127** N (%)	**Bilateral Support N=104** N (%)	**Wheelchair -Bound N=137** N (%)	**p-value** [α]
**Infections resulting in:**						
Hospitalization	79 (6.5%)*	17 (2.7%)*	10 (7.1%)*	13 (9.6%)*	39 (21.2%)*	**<0.001**
IV antibiotics (without hospitalization)	6 (0.6%)	2 (0.3%)	0 (0.0%)	2 (1.9%)	2 (1.5%)	0.078
Extended dosing antibiotics	15 (1.5%)	5 (0.8%)	2 (1.6%)	2 (1.9%)	6 (4.4%)	**0.016**
**Infusion reaction requiring hospitalization**	0 (0.0%)	0 (0.0%)	0 (0.0%)	0 (0.0%)	0 (0.0%)	-
**Malignant Cancer**	9 (0.9%)	4 (0.6%)	0 (0.0%)	1 (1.0%)	4 (2.9%)	0.066
**New autoimmune disease diagnosis**	6 (0.6%)	5 (0.8%)	0 (0.0%)	0 (0.0%)	1 (0.7%)	0.916
**Thromboembolic event (Non-superficial)**	8 (0.8%)	0 (0.0%)	3 (2.4%)	1 (1.0%)	4 (2.9%)	**0.003**
**Lymphopenia**						
<500 cells/mm3	48 (5.1%)	23 (4.0%)	10 (8.2%)	5 (5.0%)	10 (7.9%)	0.098
<200 cells/mm3	2 (0.2%)	0 (0.0%)	2 (1.6%)	0 (0.0%)	0 (0.0%)	**0.029**
**Neutropenia**						
<1000 cells/mm3	14 (1.5%)	9 (1.6%)	2 (1.6%)	2 (2.0%)	1 (0.8%)	0.888
<500 cells/mm3	11 (1.2%)	7 (1.2%)	2 (1.6%)	1 (1.0%)	1 (0.8%)	0.916
**Low IgG Values**						
<500 mg/dL	38 (5.2%)	18 (3.9%)	7 (7.5%)	7 (8.1%)	6 (6.4%)	0.175
<300 mg/dL	8 (1.1%)	5 (1.1%)	1 (1.1%)	1 (1.2%)	1 (1.1%)	1.000
**Mortality**	14 (1.4%)	2 (0.3%)	0 (0.0%)	3 (2.9%)	9 (6.6%)	**<0.001**

Bold p-values indicate p>0.05 and are considered statistically significant. N: number of events occurred; %: percent of patients who experienced event.

* Sixty-five patients (17 no device; 9 unilateral support; 10 bilateral support; 29 wheelchair-bound) were hospitalized a total of 79 times (17 no device; 10 unilateral support; 13 bilateral support; 39 wheelchair-bound). Nine patients (0 no device; 1 unilateral support; 2 bilateral support; 6 wheelchair-bound) were hospitalized multiple times due to infections.

[α] Comparing all disability levels; Significance indicates outcomes are dependent on disability level

Subgroup Analysis- By Immunosuppression/Chemotherapy History

Table S14. Percentage of patients who experienced a serious safety event on rituximab/ocrelizumab by immunosuppression/chemotherapy history.

		**Immunosuppression/Chemotherapy History^**			
	**Total** N (%)	**No history N= 841** N (%)	**Prior treatment N= 106** N (%)	**Current treatment N=26** N (%)	**p-value** [α]
**Infections resulting in:**					
Hospitalization	79 (6.5%)*	52 (5.6%)	25 (14.8%)	2 (7.1%)	**0.001**
IV antibiotics (without hospitalization)	6 (0.6%)	4 (0.5%)	2 (1.9%)	0 (0.0%)	0.278
Extended dosing antibiotics	15 (1.5%)	11 (1.3%)	4 (3.7%)	0 (0.0%)	0.135
**Infusion reaction requiring hospitalization**	0 (0.0%)	0 (0.0%)	0 (0.0%)	0 (0.0%)	-
**Malignant Cancer**	9 (0.9%)	9 (1.1%)	0 (0.0%)	0 (0.0%)	0.699
**New autoimmune disease diagnosis**	6 (0.6%)	6 (0.6%)	0 (0.0%)	0 (0.0%)	1.000
**Thromboembolic event (Non-superficial)**	8 (0.8%)	4 (0.5%)	2 (1.9%)	2 (7.1%)	**0.005**
**Lymphopenia**					
<500 cells/mm3	48 (5.1%)	31 (3.9%)	11 (11.0%)	5 (20.0%)	**<0.001**
<200 cells/mm3	2 (0.2%)	0 (0.0%)	1 (1.0%)	1 (4.0%)	**0.007**
**Neutropenia**					
<1000 cells/mm3	14 (1.5%)	10 (1.3%)	2 (2.0%)	2 (8.0%)	0.057
<500 cells/mm3	11 (1.2%)	8 (1.0%)	2 (2.0%)	1 (4.0%)	0.122
**Low IgG Values**					
<500 mg/dL	38 (5.2%)	26 (4.1%)	10 (12.5%)	2 (10.0%)	**0.006**
<300 mg/dL	8 (1.1%)	1 (0.16%)	6 (7.5%)	1 (5.0%)	**<0.001**
**Mortality**	14 (1.4%)	13 (1.5%)	1 (0.9%)	0 (0.0%)	1.000

Bold p-values indicate p>0.05 and are considered statistically significant. N: number of events occurred; %: percent of patients who experienced event.

^Excluding MS therapies of natalizumab, dimethyl fumarate and fingolimod.

* Sixty-five patients (47 no history; 16 prior treatment; 2 current treatment) were hospitalized a total of 79 times (47 no history; 25 prior treatment; 2 current treatment). Nine patients (4 no history; 5 prior treatment; 0 current treatment) were hospitalized multiple times due to infections.

[α] Comparing no, prior and current treatment; Significance indicates outcomes are dependent on immunosuppression/chemotherapy History
